# Supplementary material for: Who Benefits Most from Positive Psychological Interventions? Predictors and Moderators of Well-Being Outcomes in Severe Mental Health Conditions
Source: Healthcare (Basel). 2025 Aug 13;13(16):1988. doi: 10.3390/healthcare13161988 (PMC12385244; doi:10.3390/healthcare13161988)
Supplement: Supplementary file 1 [file healthcare-13-01988-s001.zip › healthcare-3758786-supplementary.pdf]

Table S1. *Sociodemographic and clinical baseline characteristics as a predictors and Moderators Well-Being Changes*

	b (95% CI), P		Effect Size	
Variables	Main effect, prediction	Interaction with treatment, moderation	Predictor effect size R^2	Moderator effect size ΔR^2
Gender (reference women)				
-Autonomy	-0.45 (-3.1 to -2.19), 0.73	0.96 (-2.86 to 4.8), 0.62	0.02	0.002
-Self-acceptance	-1.23 (-3.8 to 1.3), 0.35	3.42 (-0.34 to 7.1), 0.07	0.05	0.02
-Relationships others	-0.74 (-3.6 to 2.1), 0.61	0.68 (-3.4 to 4.8), 0.74	0.04	0.0009
-Environmental mastery	-0.06 (-2.2 to 2.1), 0.95	0.74 (-3.6 to 5.1), 0.73	0.03	0.001
-Purpose in life	-1.33 (-4.2 to 1.5), 0.36	3.52 (-0.70 to 7.7), 0.10	0.02	0.02
-Personal growth	-0.32 (-3.1 to 2.4), 0.81	2.43 (-1.5 to 6.4), 0.23	0.01	0.01
-Satisfaction with life	-1.88 (-4.5 to 0.73), 0.15	1.53 (-2.2 to 5.3), 0.42	0.02	0.005
Age				
-Autonomy	-0.05 (-0.18 to 0.08), 0.44	0.01 (-0.18 to 0.21) 0.88	0.03	0.0002
-Self-acceptance	0.05 (-0.08 to 0.18), 0.46	-0.05 (-0.25 to 0.14), 0.60	0.03	0.002
-Relationships others	0.007 (-0.13 to 0.15), 0.91	0.08 (-0.12 to 0.30), 0.41	0.01	0.005
-Environmental mastery	0.03 (-0.07 to 0.15), 0.51	0.15 (-0.07 to 0.37), 0.19	0.05	0.01
-Purpose in life	-0.002 (-0.15 to 0.14), 0.97	0.10 (-0.12 to 0.32), 0.37	0.01	0.007
-Personal growth	-0.14 (-0.27 to -0.003), 0.04	0.32 (.11 to 0.52), 0.002*	0.07	0.07
-Satisfaction with life	-0.01 (-0.15 to 0.11), 0.81	-0.09 (-0.28 to 0.10), 0.36	0.02	0.007
Civil status (reference single)				
-Autonomy	-0.21 (-1.84 to 1.41), 0.79	-1.76 (-5.2 to 1.72), 0.31	0.03	0.008
-Self-acceptance	0.93 (-0.68 to 2.56), 0.25	-1.5 (-4.9 to 1.9), 0.39	0.04	0.006
-Relationships others	1.19 (-0.54 to 2.9), 0.17	-3.5 (-7.2 to 0.22), 0.06	0.03	0.03
-Environmental mastery	-1.4 (-3.4 to 0.46), 0.13	-2.98 (-6.9 to 0.98), 0.13	0.06	0.01
-Purpose in life	0.37 (-1.4 to 2.1), 0.68	-0.48 (-4.4 to 3.4), 0.80	0.003	0.0005
-Personal growth	-0.47 (-2.1 to 1.2), 0.57	-3.16 (-6.7 to 0.45), 0.08	0.04	0.02
-Satisfaction with life	0.35 (-1.2 to 1.9), 0.65	-0.08 (-3.5 to 3.3), 0.95	0.004	0
Education (reference low level)				
-Autonomy				
-Self-acceptance	-0.26 (-2.2 to 1.7), 0.79	1.18 (-1.4 to 3.78), 0.37	0.02	0.007
-Relationships others	0.92 (-1.04 to 2.9), 0.35	-0.67 (-3.2 to 1.9), 0.60	0.03	0.002
-Environmental mastery	-1 (-3.1 to 1.1), 0.35	0.19 (-2.6 to 3.01), 0.89	0.01	0.0002
-Purpose in life	0.69 (-0.80 to 2.2), 0.35	0.42 (-2.5 to 3.4), 0.78	0.04	0.0007
-Personal growth	0.55 (-1.6 to 2.7), 0.61	-2.17 (-5.03 to 0.70), 0.13	0.02	0.02
-Satisfaction with life	0.11 (-1.9 to 2.2), 0.91	-0.75 (-3.5 to 2), 0.58	0.005	0.002
	0.45 (-1.5 to 2.4), 0.64	-0.65 (-3.2 to 1.9), 0.61	0.005	0.002
Employment status (unemployed)				
-Autonomy				
-Self-acceptance	-2.32 (-4.4 to -0.19), 0.03*	2.28 (-0.88 to 5.45), 0.15	0.06	0.01
-Relationships others	1.07 (-1.1 to 3.2), 0.32	-1.22 (-4.4 to 2.01), 0.45	0.03	0.004
-Environmental mastery	0.78 (-1.5 to 3.1), 0.50	-1.03 (-4,5 to 2.4), 0.56	0.004	0.003
-Purpose in life	-1.3 (-3.1 to 0.53), 0.16	-2.2 (-5.8 to 1.4), 0.23	0.06	0.01
-Personal growth	-0.72 (-3.1 to 1.6), 0.55	1.51 (-2.07 to 5.1), 0.40	0.008	0.006
-Satisfaction with life	1.6 (-0.68 to 3.8), 0.16	-2.52 (-5.9 to 0.86), 0.14	0.02	01
	0.58 (-1.5 to 2.7), 0.58	-1.2 (-4.3 to 1.9), 0.45	0.009	0.005
	b (95% CI), P		Effect Size	
Variables	Main effect, prediction	Interaction with treatment, moderation	Predictor effect size R^2	Moderator effect size ΔR^2
Main diagnosis (reference psychosis)				
-Autonomy	-1.77 (-4.9 to 1,4), 0.27	2.06 (-2.6 to 6.7), 0.38	0.02	0.007
-Self-acceptance	-2.33 (-5.2 to 0.60), 0.11	1.37 (-2.9 to 5.6), 0.52	0.08	0.003
-Relationships others	-3.9 (-7.2 to -0.56), 0.02*	4.72 (-0.16 to 9.6), 0.05	0.05	0.03
-Environmental mastery	-1.15 (-4.6 to 2.3), 0.50	-0.56 (-5.6 to 4.5), 0.82	0.03	0.0005
-Purpose in life	-4.2 (-7.6 to -0.91), 0.01*	2.91 (-1.9 to 7.7), 0.23	0.07	0.01
-Personal growth	0.12 (-3.2 to 3.5), 0.94	-0.29 (-5.2 to 4.6), 0.90	0.002	0.0001
-Satisfaction with life	0.82 (-2.4 to 4.05), 0.61	0.43 (-4.2 to 5.1), 0.85	0.09	0.0003
Nº therapy sessions per week				

-Autonomy	0.78 (-2.18 to 3.74), 0.60	-0.70 (-4.03 to 2,62), 0.67	0.02	0.001
-Self-acceptance	-3.7 (-6.6 to -0.80), 0.01	4.49 (1.2 to 7.7), 0.007*	0.09	0.06
-Relationships others	2.1 (-1.05 to 5,2), 0.19	-2.34 (-5.8 to 1.1), 0.19	0.01	0.01
-Environmental mastery	0.84 (-1.07 to 2.7), 0.38	1.25 (-2.5 to 5.03), 0.51	0.05	0.003
-Purpose in life	-0.48 (-3.7 to 2.8), 0.77	-0.55 (-4.2 to 3.1), 0.76	0.01	0.0008
-Personal growth	-1.58 (-4.7 to 1.5), 0.31	2.41 (-1.08 to 5.9), 0.17	0.01	0.01
-Satisfaction with life	0.34 (-2.5 to 3.2), 0.81	-1.17 (-4.4 to 2.06), 0.47	0.01	0.004
Age at first symptom				
-Autonomy	-0.03 (-0.19 to 0.13), 0.69	0.11 (-0.18 to 0.41), 0.45	0.008	0.006
-Self-acceptance	-0.12 (-0.27 to 0.03), 0.11	0.02 (-0.25 to 0.29), 0.88	0.08	0.0002
-Relationships others	-0.05 (-0.22 to 0.11), 0.49	0.14 (-0.17 to 0.44), 0.37	0.01	0.009
-Environmental mastery	0.03 (-0.14 to 0.21), 0.69	0.10 (-0.22 to 0.43), 0.53	0.03	0.004
-Purpose in life	-0.02 (-0.21 to 0.16), 0.80	-0.12 (-0.46 to 0.21), 0.46	0.02	0.006
-Personal growth	-0.14 (-0.32 to 0.03), 0.12	0.24 (-0.08 to 0.57), 0.14	0.03	0.02
-Satisfaction with life	0.04 (-0.11 to 0.21), 0.54	-0.09 (-0.39 to 0.19), 0.50	0.007	0.005
Alcohol consumption (Yes)				
-Autonomy	-1.87 (-4 to 0.25), 0.08	2.46 (-0.49 to 5.4)., 0.10	0.04	0.02
-Self-acceptance	-0.60 (-2.7 to 1.5), 0.57	-0.92 (-3.9 to 2.04), 0.53	0.04	0.003
-Relationships others	-0.68 (-3.05 to 1.6), 0.56	0.56 (-2.7 to 3.8), 0.73	0.003	0.001
-Environmental mastery	-0.10 (-2.3 to 2.2), 0.93	-3.04 (-6.2 to 0.183), 0.06	0.09	0.03
-Purpose in life	-0.56 (-2.6 to 1.5), 0.60	-2.77 (-5.7 to 0.17), 0.06	0.10	0.02
-Personal growth	-0.22 (-2.4 to 2), 0.84	-3 (-6.08 to 0.07), 0.05	0.07	0.03
-Satisfaction with life	-1.54 (-3.6 to 0.53), 0.14	-0.39 (-3.2 to 2.4), 0.78	0.05	0.0006

Table S2. *Psychopathological symptoms and Life Satisfaction as predictors and moderators of Well-Being Changes*

	b (95% CI), *P*		Effect Size	
Variables	Main effect, prediction	Interaction with treatment, moderation	Predictor effect size R^2	Moderator effect size ΔR^2
Somatization				
-Autonomy	1.78 (0.20 to 3.3), 0.02	-2.89 (-5.1 to -0.63), 0.01*	0.07	0.05
-Self-acceptance	0.86 (-0.73 to 2.4), 0.28	-0.96 (-3.2 to 1.3), 0.40	0.04	0.005
-Relationships others	1.13 (-0.61 to 2.8), 0.20	-1.45 (-3.9 to 1.05), 0.25	0.01	0.01
-Environmental mastery	1.56 (-0.25 to 3.4), 0.09	-2.3 (-4.9 to 0.32), 0.15	0.06	0.02
-Purpose in life	-1.28 (-0.48 to 3.06), 0.15	-0.20 (-2.7 to 2.3), 0.88	0.03	0.0002
-Personal growth	1.08 (-0.60 to 2.7), 0.20	-1.68 (-4.1 to 0.73), 0.16	0.01	0.01
-Satisfaction with life	0.85 (-0.75 to 2.4), 0.29	-0.19 (-2.5 to 2.1), 0.86	0.01	0.0002
Obsessive–compulsive				
-Autonomy	0.77 (-0.63 to 2.1), 0.28	-0.32 (-2.4 to 1.8), 0.76	0.03	0.0008
-Self-acceptance	1.03 (-0.36 to 2.4), 0.14	-0.63 (-2.7 to 1.4), 0.55	0.05	0.003
-Relationships others	0.04 (-1.5 to 1.5), 0.95	-0.14 (-2.4 to 2.1), 0.90	0.0001	0.0001
-Environmental mastery	0.50 (-1.1 to 2.1), 0.53	-1.8 (-4.2 to 0.60), 0.14	0.05	0.01
-Purpose in life	1.18 (-0.37 to 2.7), 0.13	-0.90 (-3.2 to 1.4), 0.44	0.02	005
-Personal growth	0.61 (-0.86 to 2.08), 0.41	-1.77 (-3.9 to 0.43), 0.11	0.02	0.02
-Satisfaction with life	-0.40 (-1.8 to 1.01), 0.57	0.77 (-1.3 to 2.9), 0.47	0.005	0.004
Interpersonal sensitivity				
-Autonomy	0.23 (-1.1 to 1.6), 0.74	-0.67 (-2.8 to 1.5), 0.54	0.02	0.003
-Self-acceptance	1.68 (0.34 to 3.02), 0.01*	-1.06 (-3.1 to 1.04), 0.32	0.08	0.008
-Relationships others	-0.05 (-1.5 to 1.4), 0.94	-0.16 (-2.5 to 2.2), 0.89	0.005	0.0002
-Environmental mastery	1.01 (-0.55 to 2.5), 0.20	-2.01 (-4.4 to 0.44), 0.10	0.05	0.02
-Purpose in life	0.99 (-0.53 to 2.5), 0.20	-0.25 (-2.6 to 2.1), 0.83	0.02	0.0004
-Personal growth	1.15 (-0.27 to 2.6), 0.11	-1.91 (-4.1 to 0.33), 0.09	0.02	0.02
-Satisfaction with life	-0.27 (-1.6 to 1.1), 0.70	0.91 (-1.2 to 3.08), 0.40	0.007	0.006
Depression				
-Autonomy	0.70 (-0.76 to 2.1), 0.34	-0.18 (-2.3 to 1.9), 0.86	0.03	0.0002
-Self-acceptance	1.15 (-0.29 to 2.6), 0.11	-1.13 (-3.2 to 0.94), 0.28	0.05	0.01
-Relationships others	0.16 (-1.4 to 1.7), 0.84	-0.28 (-2.5 to 2.01), 0.80	0.0005	0.0005
-Environmental mastery	0.87 (-0.80 to 0.2.5), 0.30	-1.27 (-3.6 to 1.1), 0.29	0.04	0.01
-Purpose in life	0.86 (-0.74 to 2.4), 0.28	0.54 (-1.7 to 2.8), 0.64	0.03	0.002
-Personal growth	0.82 (-0.70 to 2.3), 0.28	-2.03 (-4.2 to 0.15), 0.06	0.03	0.03
-Satisfaction with life	-1.02 (-2.4 to 0.42), 0.16	1.57 (-0.51 to 3.6), 0.14	0.02	0.02
Anxiety				

-Autonomy	0.51 (-0.84 to 1.8), 0.45	-0.72 (-2.9 to 1.4), 0.51	0.02	0.003
-Self-acceptance	0.90 (-0.43 to 2.2), 0.18	-0.99 (-3.1 to 1.1), 0.36	0.04	0.007
-Relationships others	-0.11 (-1.5 to 1.3), 0.88	-0.13 (-2.5 to 2.2), 0.91	0.0008	0.0001
-Environmental mastery	0.41 (-1.1 to 1.9), 0.60	-1.98 (-4.4 to 0.48), 0.11	0.05	0.02
-Purpose in life	0.77 (-0.72 to 2.2), 0.30	0.38 (-2.02 to 2.7), 0.75	0.02	0.0008
-Personal growth	0.13 (-1.2 to 1.5), 0.85	-1.6 (-3.8 to 0.67), 0.16	0.02	0.01
-Satisfaction with life	-0.38 (-1.7 to 0.97), 0.57	0.85 (-1.3 to 3.03), 0.43	0.006	0.005

	b (95% CI), *P*		Effect Size	
Variables	Main effect, prediction	Interaction with treatment, moderation	Predictor effect size R^2	Moderator effect size ΔR^2
Hostility				
-Autonomy	0.79 (-0.84 to 2.4), 0.33	-1.95 (-4.2 to 0.38), 0.10	0.04	0.02
-Self-acceptance	1.25 (-0.36 to 2.8), 0.12	-1.75 (-4.07 to 0.56), 0.13	0.05	0.01
-Relationships others	1.56 (-0.19 to 3.3), 0.08	-2.71 (-5.2 to -0.20), 0.03*	0.04	0.03
-Environmental mastery	1.04 (-0.82 to 2.9), 0.27	-1.65 (-4.3 to 1.01), 0.22	0.04	0.01
-Purpose in life	0.68 (-1.1 to 2.5), 0.45	0.65 (-1.9 to 3.2), 0.61	0.02	0.002
-Personal growth	1.38 (-0.29 to 3.06), 0.10	-3.3 (-5.7 to -0.91), 0.007*	0.06	0.06
-Satisfaction with life	1.20 (-0.43 to 2.8), 0.14	-1.00 (-3.3 to 1.3), 0.39	0.02	0.006
Phobic anxiety				
-Autonomy	0.45 (-0.85 to 1.7), 0.49	-1.54 (-3.7 to 0.61), 0.15	0.03	0.01
-Self-acceptance	0.54 (-0.75 to 1.8), 0.41	-0.43 (-2.5 to 1.7), 0.69	0.04	0.001
-Relationships others	-0.02 (-1.4 to 1.4), 0.97	0.27 (-208 to 2.6), 0.82	0.0006	0.0004
-Environmental mastery	0.81 (-0.67 to 2.3), 0.28	-1.02 (-3.5 to 1.4), 0.41	0.04	0.005
-Purpose in life	0.80 (-0.64 to 2.2), 0.27	-0.63 (-3.03 to 1.7), 0.60	0.01	0.002
-Personal growth	-0.27 (-1.6 to 1.1), 0.69	-0.35 (-2.6 to 1.9), 0.76	0.005	0.0008
-Satisfaction with life	-1.13 (-2.4 to 0.14), 0.08	0.82 (-1.31 to 2.9), 0.45	0.02	0.005
Paranoid ideation				
-Autonomy	0.08 (-1.3 to 1.4), 0.90	-1.05 (-3.1 to 1.05),.32	0.03	0.008
-Self-acceptance	1.19 (-0.18 to 2.5), 0.08	-1.82 (-3.9 to 0.25), 0.08	0.06	0.23
-Relationships others	0.05 (-1.4 to 1.5), 0.94	-0.20 (-2.5 to 2.1), 0.86	0.0003	0.0003
-Environmental mastery	1.2 (-0.38 to 2.7), 0.13	-1.9 (-4.3 to 0.48), 0.11	0.05	0.02
-Purpose in life	0.51 (-1.02 to 2.05), 0.50	0.56 (-1.7 to 2.9), 0.63	0.01	0.002
-Personal growth	0.60 (-0.85 to 2.05), 0.41	-1.71 (-3.9 to 0.48), 0.12	0.02	0.02
-Satisfaction with life	-0.63 (-2.02 to 0.76), 0.37	0.61 (-1.5 to 2.72), 0.56	0.007	0.003
Psychoticism				
-Autonomy	0.30 (-1.2 to 1.9), 0.70	-0.34 (-2.6 to 1.9), 0.76	0.02	0.0008
-Self-acceptance	1.31 (-0.24 to 2.8), 0.09	-1.42 (-3.6 to 0.81), 0.21	0.05	0.01
-Relationships others	-0.01 (-1.7 to 1.7), 0.98	0.30 (-2.1 to 2.7), 0.81	0.001	0.005
-Environmental mastery	0.94 (-0.86 to 2.7), 0.30	-1.42 (-4.02 to 1.1), 0.27	0.04	0.01
-Purpose in life	1.11 (-0.63 to 2.8), 0.21	-0.55 (-3.06 to 1.9), 0.66	0.01	0.001
-Personal growth	0.81 (-0.84 to 2.4), 0.33	-1.32 (-3.7 to 1.06), 0.27	0.01	0.01
-Satisfaction with life	-0.26 (-1.8 to 1.3), 0.73	1.44 (-0.81 to 3.7), 0.20	0.02	0.01
SCL-90 Total (Severity)				
-Autonomy	0.87 (-0.85 to 2.6), 0.32	-1.32 (-3.9 to 1.2), 0.31	0.03	0.008
-Self-acceptance	1.50 (-0.19 to 3.2), 0.08	-1.56 (-4.1 to 0.97), 0.22	0.06	0.01
-Relationships others	0.28 (-1.5 to 2.1), 0.76	-0.58 (-3.4 to 2.2), 0.68	0.001	0.001
-Environmental mastery	1.24 (.70 to 3.1). 0.21	-2.37 (-5.3 to 0.54), 0.11	0.05	0.02
-Purpose in life	1.24 (-0.64 to 3.1), 0.19	-0.11 (-2.9 to 2.7), 0.93	0.02	0.0001
-Personal growth	0.96 (-0.82 to 2.7), 0.28	-2.38 (-5.06 to 0.30), 0.08	0.02	0.02
-Satisfaction with life	-0.47 (-2.2 to 1.2), 0.58	1.14 (-1.4 to 3.7), 0.38	0.007	0.006
SWLS				
-Autonomy	0.05 (-0.13 to 0.25), 0.56	-0.10 (-0.37 to 0.16), 0.43	0.02	0.005
-Self-acceptance	0.04 (-0.14 to 0.24), 0.61	-0.21 (-0.47 to 0.04), 0.10	0.06	0.02
-Relationships others	0.10 (-0.05 to 0.24), 0.20	-0.14 (-0.43 to 0.15), 0.34	0.02	0.007
-Environmental mastery	0.20 (-0.005 to.42), 0.05	-0.43 (-0.73 to -0.13), 0.004*	0.10	0.06
-Purpose in life	0.13 (-0.08 to 0.34), 0.21	-0.24 (-0.53 to 0.05), 0.11	0.02	0.02
-Personal growth	-0.03 (-0.23 to 0.17), 0.75	0.08 (-0.20 to 0.36), 0.57	0.002	0.002
